# “Busting the hidden curriculum” a realist and innovative perspective to foster professional behaviors

**DOI:** 10.3389/fmed.2024.1484058

**Published:** 2024-12-04

**Authors:** Shaista Salman Guraya, Grainne P. Kearney, Frank Doyle, Asil Sadeq, Abdelsalam Bensaaud, Eric Clarke, Mark Harbinson, Aine Ryan, Mary Smyth, Sinead Hand, Fiona Boland, Salman Yousuf Guraya, Denis W. Harkin

**Affiliations:** ^1^Institute of Learning, Mohammad Bin Rashid University of Medicine and Health Sciences, Dubai, United Arab Emirates; ^2^School of Medicine, Dentistry and Biomedical Sciences, Queens University Belfast, Belfast, United Kingdom; ^3^Department of Health Psychology, School of Population Health, RCSI University of Medicine and Health Sciences, Dublin, Ireland; ^4^Centre for Professionalism in Medicine and Health Sciences, Faculty of Medicine and Health Sciences, RCSI University of Medicine and Health Sciences, Dublin, Ireland; ^5^Data Science Centre, School of Population Health, RCSI University of Medicine and Health Sciences, Dublin, Ireland; ^6^Clinical Sciences Department, College of Medicine, University of Sharjah, Sharjah, United Arab Emirates

**Keywords:** hidden curriculum, professional behaviors, medical professionalism, design-based research, theory of planned behavior, mixed-methods study

## Abstract

Contemporary health professions education has long delineated the desired attributes of medical professionalism in the form of standard curricula and their role in forming professional behaviors (PBs) among aspiring doctors. However, existing research has shown the contradictory and powerful role of hidden curriculum (HC) in negatively influencing medical students’ PBs through unspoken or implicit academic, cultural, or social standards and practices. These contrasting messages of formal curricula and HC lead to discordance and incongruence in future healthcare professionals developing professional identity formation. There is little research on PB modifying educational strategies and their determinants that medical schools adopt to bust the impact of HC. Consequently, it is unclear how the right PBs can be influenced, entrenched, and inculcated in undergraduate medical students, especially in their early clinical placements. The lack of such insight highlights a critical gap in the literature, nudging educators to take a realist stance to deal with this problem. Behavior psychology stresses shaping medical students’ values and beliefs as salient mediators that influence intentions to pursue future PBs. Curiosity prevails about what would guide the educational interventions to target this behavior change. To help understand this concept, we present our design-based innovative perspective about PROfessionalism in Partnership for Education Research (PROPER) shaped by pluralistic theoretical models in the context of two European medical schools with diverse medical students, highlighting its non-parochial and transferable nature.

## 1 Background and need for innovation

In delivering safe and high-quality patient care, there has been a notable realization of the importance of upholding professional standards. This has led to a call for the establishment of professional behaviors (PB) in future healthcare professionals (HCPs) through modern health professions education (HPE) (1). However, the current state of HPE not only falls short in responding to this call but also impedes progress toward the right direction in some ways. Martimianaks et al. (2), highlighted the increasing recognition of a hidden curriculum in negatively influencing PBs and professional identity formation Haferty (3, 4), described this HC-driven phenomenon as a source of implicit messages, values, and norms conveyed to learners through informal deliberations. These subtle influences can significantly shape students’ perceptions of their physician roles. This pervasive and often negative influence of HC on professional teaching in contemporary medical education is now increasingly being acknowledged (5–9).

The current state of formal curricula in HPE may explicitly outline professional expectations and competencies, while hidden ones may give conflicting messages or reinforce stereotypes and biases that negate these efforts (10, 11). For instance, in a qualitative study of medical students perceptions, Lempp and Seale (9) highlighted how senior clinicians’ role modeling of hierarchical power dynamics and negative cultures in hospitals can inadvertently promote unprofessional behaviors or attitudes among medical students and doctors in training. Therefore, HPE programs realize it is incumbent upon them to act by incorporating explicit training on professionalism and HC into the formal curriculum (12). By integrating discussions on professionalism, ethical decision-making, and the impact of organizational culture into didactic sessions, workshops, and clinical skills training, educators can raise students’ awareness of HC and may provide them with the knowledge and skills to navigate its complexities effectively (13).

However, there remains a need for fundamental changes to mitigate and counteract the impact of HC on medical professionalism (MP) by using multifaceted approaches at all levels of undergraduate medical education (9, 14, 15). Sheeran (16) reminds us that this requirement can only be met effectively using scientific methods with experimental studies to identify the exact mechanisms involved in behavioral change interventions. This includes a detailed examination of the behaviors and theories of behavioral change underpinning the educational intervention and the use of these behavioral change models to marshal cumulative factors. Behavioral change research primarily forces educators to look for who, what, when, where, and how they need to do differently to motivate learners to adopt, adapt or pursue certain behaviors. This starts with a detailed analysis of identified behaviors and highlights its relationship in the wider individual or social behavioral networks (17). This, in turn, leads to the identification of professional, financial, organizational, or regulatory factors and self, peers, supervisors, family, and social media actors, which may influence the behaviors in question (18).

At this point, there remains a curiosity about what would guide the educational interventions targeting behavior change. To help us understand, a theory can provide a logical relationship between various abstract concepts that explain the world around us (19). This can help educators develop interventions that may be applied to different situations and contexts to achieve learning outcomes and behavior change (20, 21). Nevertheless, it’s crucial to note that merely identifying theoretical constructs will not serve as a magic bullet. Identification of behavioral change strategies (22, 23) to influence the identified theoretical constructs are the frequently missed pieces of the puzzle in the intervention design (12, 24). Reflecting on the fundamental “master question” in education research: *What should be taught to whom, when, and how?* we recognize that effective educational planning must address not only what and to whom but also how behavioral change strategies align with educational objectives (22, 23). Hence, the educational intervention should identify the behavior-predicting determinants (theoretical framework), behavior change strategy targeting those determinants, and the practical application keeping the target population in mind (22, 25). This alignment forms the foundation of design-based research (DBR) (26), an innovative approach that emphasizes designing, testing, and refining educational interventions in real-world settings. DBR’s pluralistic nature supports crossing theoretical and methodological boundaries, integrating theories to explore the interactions between context and behavioral mechanisms in complex environments.

In an attempt to respond to the identified gaps and the call for the promotion of PBs in future HCPs, borrowing DBR principles, we have proposed an educational intervention entitled the PROfessionalism in Partnership for Education Research (PROPER) study. PROPER uses a four-step experimental design approach, namely: (1) specifying the PBs, (2) analyzing PBs, (3) designing the intervention, and (4) measuring the change in PBs. Additionally, we incorporate a realist perspective by identifying underlying causal mechanisms and exploring how they work under specific conditions. Through this realist perspective, we aim to understand what, how, for whom, and under what circumstances complex interventions function most effectively (27). In this perspective piece, we strive to describe our PROPER educational intervention and its theoretical framework to support HPE educators and faculty who are interested in using theory-driven approaches to target PBs in MP education.

## 2 The PROPER educational intervention

The PROPER study, funded by the Higher Education Authority, North–South Research Program, was designed to foster the development of PBs among early clinical undergraduate medical students. The intervention involved theory-driven workshops for students from two distinct educational contexts on the Island of Ireland: the Royal College of Surgeons in Ireland (RCSI) University of Medicine and Health Sciences in Dublin (Republic of Ireland) and Queen’s University Belfast (QUB) in Belfast (United Kingdom). At RCSI, professionalism is taught as an explicit strand focused on personal and professional identity formation, while QUB integrates professionalism within the basic sciences curriculum, embedding it seamlessly rather than through a dedicated module. Importantly, all participating students were at a comparable stage in their training; either in pre-clinical education or newly entering clinical placements which ensured a similar level of cognitive maturity and understanding across both groups. This consistency provided a reliable foundation for evaluating the intervention’s impact across different curricular approaches. We have reported the PROPER study using TIDiER framework (28) throughout our perspective paper (Appendix 1).

PROPER workshops were designed in such a way that they could be conducted both in-person and virtually. We conducted four 90-min structured workshops on themes relevant to the HC which were identified as important by expert consensus (nominal group process) (29, 30), involving experts in medical education and medical students. These themes were:

- Maintaining confidentiality in the clinical practice.
- Raising concerns and whistle-blowing.
- Practicing self-care and wellbeing.
- Exercising cultural sensitivity.

Box 1 entails the objectives of one of the workshops which can be used for all the identified themes.

Box 1 **Workshop objectives (DAGaRR).**

1. **Define** the terms and features associated with the concept of confidentiality at an abstract level and concrete, i.e., behavioral level.

2. **Acquire experience** in defining and articulating professional and unprofessional behaviors by describing various behaviors related to a common set of experiences illustrated in the scenarios.

3. **Gain perspectives** on how the same experience can be perceived from multiple perspectives of other individuals on a team (e.g., student, resident, faculty, family, society and council, etc.).

4. **Recognize** behaviors in yourselves and others that can be categorized unprofessional when it comes to confidentiality.

5. **Reflect** on the workshop experience in terms of your behavior and the behavior of others related to confidentiality.

**Figure 1:**
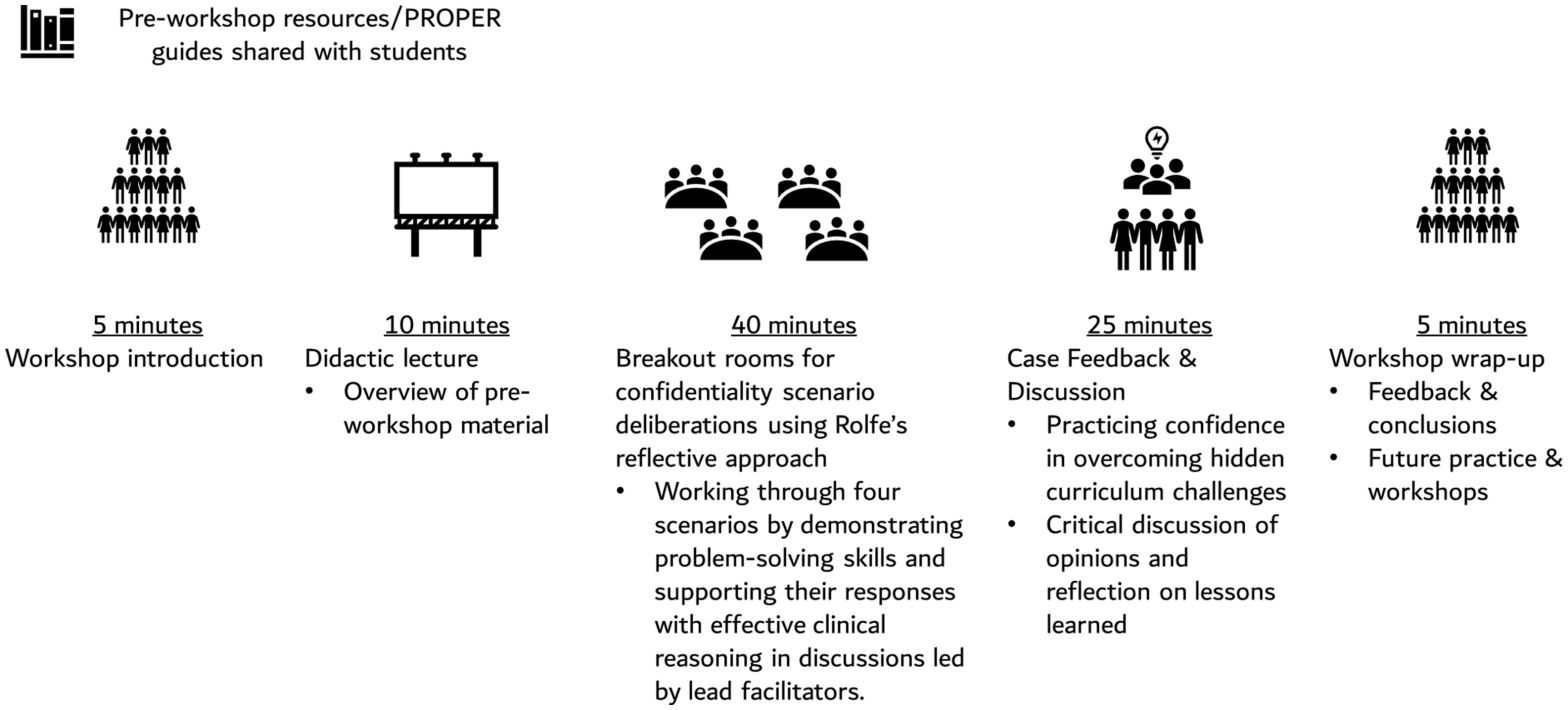
Detailed design and structure of the online PROPER intervention.

The PROPER intervention contained pre-workshop resources followed by a structured 90-min workshop as outlined in Figure 1.

## 3 Steps taken for development and implementation of innovation

**Figure 2:**
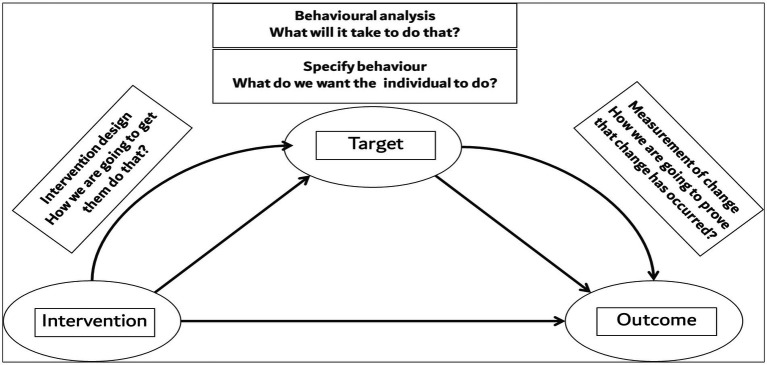
The intertwined relationship of the PROPER workshop target, educational intervention, and the outcome for measuring the changes in professional behaviors.

The salient feature of the intervention entailed identifying the desired behavior, behavioral analysis, intervention as a means of finding a mechanism to achieve the target behavior, and the perceived outcome of measuring the expected change in PBs (Figure 2).

### 3.1 Step 1: Specify the behavior – what do we want the students to do?

The influence of HC on the PBs of undergraduate medical students was the driving force. In the realm of HC, Haferty has proposed various areas that need to be safeguarded (3, 4). However, keeping the clinical context of participating institutions in mind, we identified four essential areas for PBs: maintaining confidentiality, exercising cultural sensitivity, practicing self-care, and raising concerns. To do that, we employed the nominal group process approach (29, 30), which is an expert panel method to reach a consensus on the most important PBs to address within HC in our PROPER study. This method is being used across multiple fields, particularly in healthcare research and intervention, and benefits from true expert opinion and time efficiency. A group of 8 subject-matter medical educators, practicing physicians, experts, and an educational psychologist individually proposed themes and then facilitated group discussions through a series of moderation. DWH ranked and agreed on priorities for the educational intervention. Iterative and thorough deliberations and discussions among research team members also helped us to finalize the scenarios for these aspects to be used in the PROPER.

### 3.2 Step 2: Behavioral analysis – what will it take to do that?

Later, we worked on the hypothesis of what would guide behavioral analysis. A growing body of literature indicates that utilizing behavioral theories can aid in pinpointing modifiable factors that can assist HCPs in modifying their behaviors to align with evidence-based healthcare practices. In shaping the educational interventions aimed at enhancing PBs among undergraduate medical students, it was imperative to anchor the approach within a robust theoretical framework (31). We looked for the available theories regarding behavior and behavior change to achieve a theory-driven approach to modifying PB. Davis et al. (32), have reported 82 behaviors and behavioral change theories, which can help outline modifiable factors that can assist HCPs in modifying their behaviors to align with desired healthcare practices.

The PROPER study has focused on expectancy-value social cognition models that highlight the intentional, reflective factors of behavior through the theory of planned behavior (TPB) (33). TPB proposes that behavioral intentions are the most reliable predictor of behaviors, which are influenced by three main factors (1): attitudes toward the behavior, which are based on beliefs about the consequences of the behavior (2); subjective norms, which are rooted in beliefs about normative expectations of influential individuals); and (3) perceived behavioral controls which derive from beliefs about factors facilitating or hindering behavior. The strength of TPB lies in the explicit relationship of various conceptual constructs and their relationship in influencing behaviors (34, 35). For instance, in the medical field, TPB suggests that an HCP’s intention to engage in a specific behavior, such as adhering to clinical guidelines (36), disclosing medical errors (37) or intention to be professional in the digital world (24) is shaped by their beliefs about the behavior, social influences, and the perceived ability to enact the behavior.

**Figure 3:**
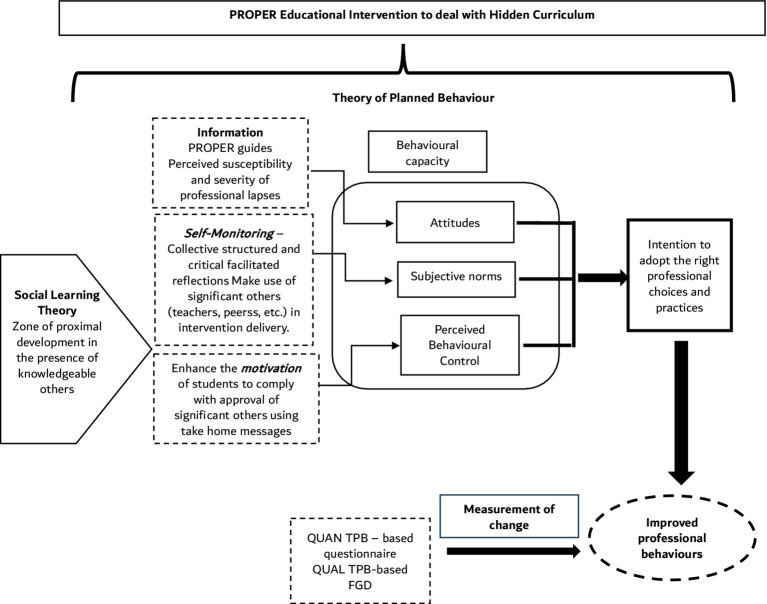
Theoretical Framework for Improving Professional Behavior Development in PROPER highlighting the interaction between the Social Learning Theory and Theory of Planned Behavior. PROPER: Professionalism in Partnership for Education Research Study; QUAN: Quantitative; QUAL; Qualitative; TPB: theory of planned behavior; FGD: Focus Group Discussion.

Another important predominant theory that underpins the PROPER study is the social cognitive theory (SCT) by Bandura (12, 38) which underpins contemporary educational practices by emphasizing the role of observation, imitation, and interaction in the acquisition and reinforcement of behaviors (38). In the context of HPE, the SCT enacts that students learn not only from didactic instruction but also from observing role models, engaging in collaborative activities, and participating in group discussions (12). Self-efficacy (39), central to SCT, is acquired through socialization in the communities of practice leading to a situated cognitive enhancement (Figure 2). Godin and colleagues’ systematic review has highlighted that TPB (33) and SCT (12, 38) have been the predominant focus for predicting HCPs’ behaviors to date, with a focus on PBs (40). Informed by the published literature, the PROPER study supported the adoption of TPB and SCT as theories of our choice (41). Figure 3 describes the collective PROPER theoretical framework.

**Figure 4:**
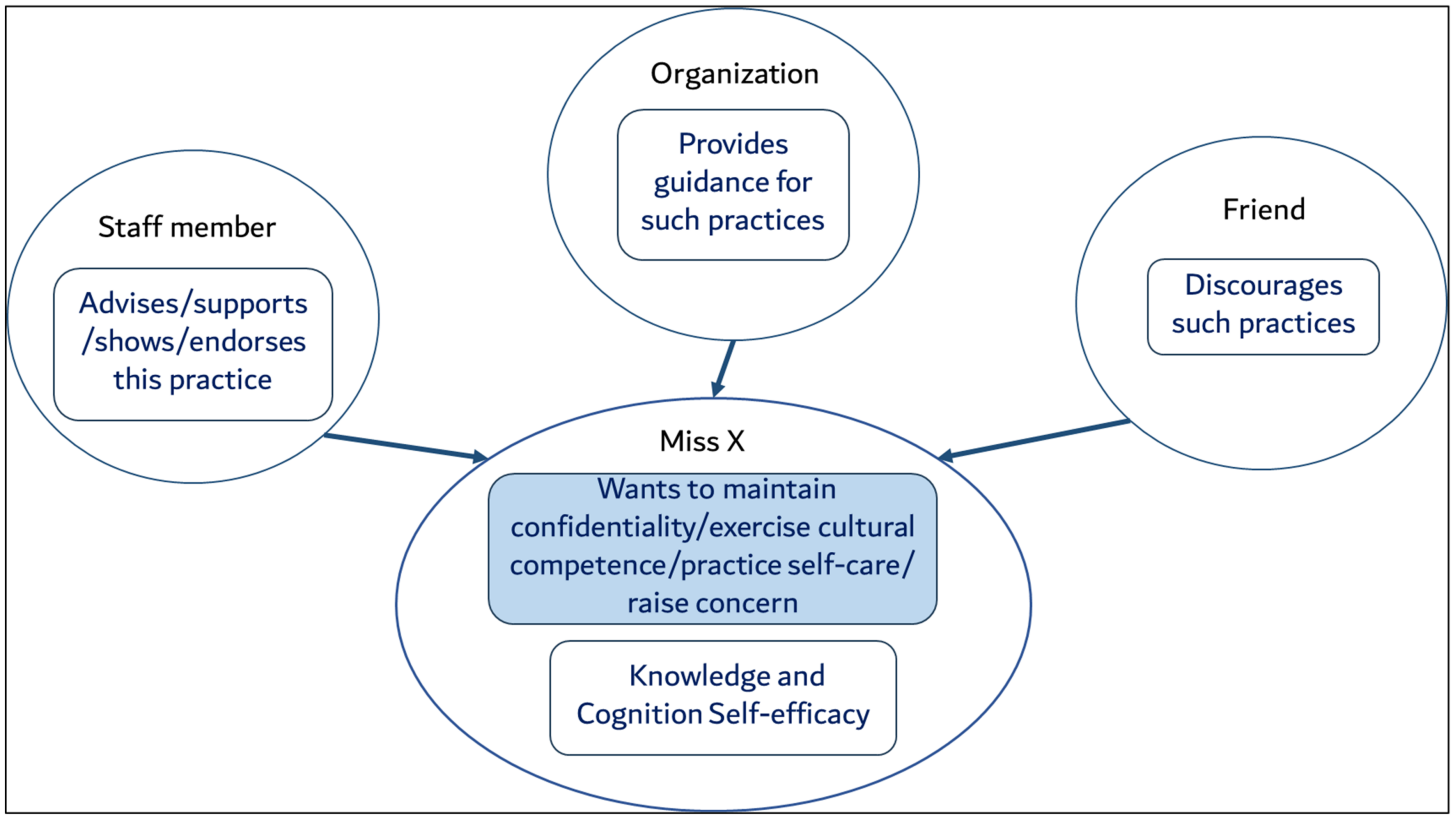
Influencing factors and actors for the behavioral analysis.

Using the TPB and SCT, we identified and signposted the modifiable factors that can be leveraged while designing interventions for behavioral changes (42). The identified four key areas were found to be occurring in a social setting, where each behavior is within the network of behaviors within each person and each person in turn within the network of other people. By adopting the behavioral analysis by Sheeran et al., we used a combination of TPB and SCT as shown in Figure 4, which illustrates the number of actors are playing their roles for Miss X’s knowledge, cognition and self-efficacy. The PROPER study however, is targeted toward changing Miss X’s behavior only.

### 3.3 Step 3: Interventional design – how will we get them to do that?

In designing the PROPER intervention, we adopted a DBR approach (26), which emphasizes the iterative development and testing of educational interventions within real-world contexts. Informed by DBR approach, we employed a pluralistic use of theories and methodologies, which allowed us to cross theoretical and methodological boundaries to address the complexity of behavioral change in healthcare education. We thoughtfully curated a sequence of educational activities integrating the constructs of the TPB constructs within the social cognition framework and settings. However, it is important to note a significant critique highlighted by Hardeman et al. (23), TPB has often been limited to measuring process and outcome variables (such as intentions) without directly informing educational intervention design, a finding commonly seen in the published literature. In the context of HPE and MP, Archer (35), Geist (43), Shiphra (44) and Guraya et al. (24), have endorsed the use of TPB to design educational interventions but did not provide a clear roadmap. Recently, Medisauskaite et al. (45), and Rich et al. (36), evaluated a professionalism-based United Kingdom program; however, the program’s design was not embedded in theoretical underpinnings. Our DBR approach allowed us to address this limitation by combining TPB with behavioral change models. Drawing from Kok (22) and Hardeman et al. (23), we developed a taxonomy of strategies to increase behavioral capacity within the intervention. Specifically, *‘Information’* was used to influence attitudes, *‘self-affirmation’* to shape subjective norms, and *‘motivation’* and *‘role modeling’* to enhance perceived behavioral control. This DBR-guided design allowed us to move beyond traditional TPB applications, providing a structured yet adaptable roadmap for developing theory-driven educational interventions. The following sections elaborate on our chosen strategies in detail, explaining how each component of the intervention aligns with our goals for advancing PBs in healthcare.

#### 3.3.1 ‘Information’—attitudes

Information has been used widely in various health behavior modification interventions (46). In our work, the modification of attitudes toward PBs was targeted using a multifaceted approach where participants understood the perceived susceptibility and severity of professional lapses. Our PROPER study utilized evidence-based information and case studies to highlight the potential consequences of unprofessional conduct, emphasizing the importance of upholding ethical standards in clinical practice. Regarding evidence-based information, resources in the form of PROPER study guides were developed collaboratively by HPE experts, clinicians and students within the PROPER study group. To ensure that guidance was appropriate, a subject expert reviewed and included universal principles while medical experts in clinical practice from the Republic of Ireland (Medical Council of Ireland) and United Kingdom (General Medical Council) reviewed and discussed areas where guidance protocols agreed or differed. These resources were named PROPER Guides and were shared with the participants before workshops. By offering accessible and relevant learning materials, the PROPER study equipped students with the knowledge and skills necessary to navigate complex professional scenarios effectively.

#### 3.3.2 Self-affirmation’ using guided reflection and facilitation—subjective norms

Self-affirmation (47) is about reflection on one’s values, attributes and social relations and has been used to improve MP understanding in our recent research (12) and in improving health-related intentions (47, 48). According to ancient Greek Philosophy the famous aphorism “know thyself” refers to the process of Reflection (49), which employs *‘self-affirmation’* as a behavior change method as elucidated by Hardeman et al. (23). This concept influences SN by enhancing self-regulatory abilities, attentional bias, and judgmental confidence (50). In real-world settings, much of the applied knowledge is implicit and requires clarification and reinterpretation to advance medical practices. Reflection is recognized as a crucial skill for doctors, playing a vital role in fostering deeper and enduring learning, as well as facilitating patient-centered care during clinical interactions (51, 52). From a learning perspective, reflection serves to validate previous learning experiences or scrutinize the rationale behind our beliefs (53). Conceptually, there is a noticeable oversight in the theoretical discourse of collective reflective practice (54). In an interesting study, tackling wicked problems (55) regarding complex public health challenges, the researchers used collective reflective practices to gain an advantage from collaborative strategies for problem-solving, particularly through structured dialogs. Theoretical frameworks often adopt an individualistic perspective, failing to adequately address the collective aspect of reflective practice.

Contrary to Schön’s theory, which portrays reflection primarily as a solitary endeavor we adopted a collective reflection, recognizing it as a social process occurring within a broader learning community (54). In our PROPER study, the participants focused on the HC using Rolf’s reflective model (56, 57) for structured guided reflection (58). During the breakout room placements, firstly, students explored their own professional values and reflected on their personal experiences within healthcare settings and we used value clarification activities to support this introspective process. Secondly, students engaged in dialogs with external factors pertinent to their clinical environment, including policies, guidelines, and ethical considerations, while sharing and discussing their reflections with peers. Finally, students were encouraged to critically analyze the social theory context (micro, meso and macro levels) of healthcare, exploring how decisions and societal norms could influence current practices. Positive expectations were made available through guided and facilitated collective discussions and scenario analysis. Students gained insights into healthcare practices, fostering a deeper understanding of HC and its impact on PB and insights into others’ approvals.

#### 3.3.3 Motivation’ and role-modeling’—perceived behavioral control

In the realm of behavior change, educational intervention might aim to increase self-efficacy beliefs to resist social pressure and choose an unprofessional course of conduct. Such belief in one’s ability to enact a PB is enhanced by applying theory-based methods such as role-modeling, guided reflective practice with feedback, and reinforcement by expert facilitators. This interactive approach hinged on Bandura’s SCT (39, 58), enhanced the PROPER students’ self-efficacy and PBC by equipping them with the skills and insights necessary to navigate complex ethical challenges in clinical practice. In the PROPER workshops, we encouraged the participants to understand take-home messages by examining the factors influencing their decisions and actions by developing a deeper awareness of their motivations and intentions. Large group wrap-ups and key take-home messages strengthened the participants’ hypothetical intentions for performing the right PBs, indicating their ability to overcome cognitive dissonance in future clinical placements. It’s imperative to highlight the role of practicing physicians as facilitators (DWH, SYG, SSG, GPK, and MH) to showcase an authentic and impactful learning experience for the participants. By providing such organized and structured reflective practices, vicarious experiences, and venues where rationalization and moral justifications of everyday situations were discussed collectively, facilitated by HCPs modeling the right choices had the potential to influence the strength of self-efficacy leading to a change in actions (12, 59).

### 3.4 Step 4: Measurement of change - how will we prove that change has occurred?

A comprehensive theory-informed approach was employed to assess the impact of PROPER study. Evaluative designs based on the TPB have been used by Guraya et al. (24), Medisauskaite et al. (45), and Rich et al. (36), however, only the first two studies (24, 45) used a mixed-methods approach, while Rich et al. (36), applied only quantitative measures. Although the methodological design varied significantly in all three studies, Guraya et al. (24), and Rich et al. (36), capitalized on pre-post design. For the PROPER study, we followed the quasi-experimental without randomization design modeled by Medisauskaite et al. (45), with self-reported pre-post, and delayed post-measurements within the intervention and control group. To further enhance the explanatory power of our approach, we conducted TPB-based FGDs to understand the causal-effect relationships and capture participants’ insights on behavioral changes (24, 60).

While these data sources provide a nuanced understanding of the intervention’s effects on professional development, we recognize that behavioral change, especially in healthcare, often requires time and sustained effort (42). Measuring outcomes shortly after the intervention may not fully capture the persistence or gradual adoption of new behaviors. Future follow-up assessments could be beneficial in evaluating the long-term impact of the intervention. Additionally, we prioritized validity by validating our TPB-based questionnaire, as reported in a recent publication (61). Finally, it is pertinent to note that while SCT informed the learning and workshop design, only TPB constructs were employed in analyzing behavioral change, ensuring a focused and reliable evaluation.

## 4 Critical reflection

Our previous research work shaped our perspective (24) in the field of MP in the context of undergraduate medical education and we built on our understanding of behavioral change theories (32). In reflecting on the “master question” in education research; *What should be taught to whom, when and how?*, we recognized that effective educational planning must address not only what and to whom but also how behavioral change strategies are mapped onto educational objectives (22, 23). This perspective highlights the complexities of developing, designing, and implementing contextually appropriate educational research. This aligns with the principles of DBR, where educational interventions function as sites for systematically studying and refining learning phenomena and where the complexity of these settings gives rise to emergent insights (26).

In this light, the PROPER study is informed by design-based research. It operates within a robust theoretical framework, drawing from both Bandura’s SCT and TPB, to enhance behavioral capacity in HPE. Nevertheless, we recognize that learning cannot be fully addressed using a few theoretical frameworks or methodological approaches. Acknowledging that SCT and TPB focus on personal and social influences, we realize that broader contextual factors such as organizational support, policies, and time constraints play a significant role in healthcare settings. We encourage our HPE community to address these systemic factors in future iterations, which could expand the intervention’s relevance and predictive power.

## Data Availability

The original contributions presented in the study are included in the article/Supplementary material, further inquiries can be directed to the corresponding author/s.
